# In-Hospital versus Out-of-Hospital Pulmonary Embolism: Clinical Characteristics, Biochemical Markers and Echocardiographic Indices

**DOI:** 10.3390/jcdd11040103

**Published:** 2024-03-28

**Authors:** Christos Ballas, Lampros Lakkas, Olga Kardakari, Eftychia Papaioannou, Konstantinos C. Siaravas, Katerina K. Naka, Lampros K. Michalis, Christos S. Katsouras

**Affiliations:** Second Department of Cardiology, University Hospital of Ioannina, 45500 Ioannina, Greeceftpcavalier52@gmail.com (L.L.); olgakardakari@yahoo.gr (O.K.); efpapaioannou@hotmail.com (E.P.); siaravaskon@gmail.com (K.C.S.); drkknaka@gmail.com (K.K.N.); lamprosmihalis@gmail.com (L.K.M.)

**Keywords:** pulmonary embolism, in-hospital, out-of-hospital, echocardiogram

## Abstract

Background: A significant proportion of pulmonary embolisms (PEs) occurs in patients during hospitalisation for another reason. However, limited data regarding differences between out-of-hospital PE (OHPE) and in-hospital PE (IHPE) is available. We aimed to compare these groups regarding their clinical characteristics, biochemical markers, and echocardiographic indices. Methods: This was a prospective, single-arm, single-centre study. Adult consecutive patients with non-COVID-related PE from September 2019 to March 2022 were included and followed up for 12 months. Results: The study included 180 (84 women) patients, with 89 (49.4%) suffering from IHPE. IHPE patients were older, they more often had cancer, were diagnosed earlier after the onset of symptoms, they had less frequent pain and higher values of high sensitivity troponin I and brain natriuretic peptide levels compared to OHPE patients. Echocardiographic right ventricular (RV) dysfunction was detected in similar proportions in the 2 groups. IHPE had increased in-hospital mortality (14.6% vs. 3.3%, *p* = 0.008) and similar post-discharge to 12-month mortality with OHPE patients. Conclusions: In this prospective cohort study, IHPE differed from OHPE patients regarding age, comorbidities, symptoms, and levels of biomarkers associated with RV dysfunction. IHPE patients had higher in-hospital mortality compared to OHPE patients and a similar risk of death after discharge.

## 1. Introduction

Venous thromboembolism (VTE) is a major cause of morbidity and mortality worldwide [1,2]. Pulmonary embolism (PE) has a greater risk of death compared to deep venous thrombosis (DVT) [2]. Early diagnosis and treatment prevent myocardial injury and right ventricular (RV) dysfunction and improve survival. However, due to the non-specific symptoms and signs of the disease, misdiagnosis or delay in diagnosis is quite common. The reported mean delay ranges from 2.5 to 11.9 days according to a recent review and meta-analysis [3]. Clinical scores have been developed and validated by physicians to overcome these problems [4,5,6,7,8,9]. In any event, a suspicion about PE is a prerequisite for it. In patients suffering from PE, haemodynamic status at presentation is the major prognostic factor, while in stable patients, a combination of clinical indices [pulmonary embolism severity index (PESI)], biomarkers [elevated values of troponin (Tn), B-type Natriuretic Peptide (BNP) or N-terminal pro-BNP (NT-proBNP)], and echocardiographic indices of right ventricular dysfunction or pressure overload (RV_d/po_) have been used to identify those with higher short-term mortality [10,11,12,13,14,15,16]. 

VTE occurs in about 1% of hospitalised patients and the annual incidence of the disease is more than 100 times greater than that among community residents [17,18]. Regarding PE, about 30–40% of the total events take place in-hospital, where patients are hospitalised for another reason (in-hospital PE, IHPE) [18,19]. Previous studies have shown that laboratory or imaging testing and clinical probability assessment may have a different diagnostic accuracy in hospitalised patients with suspected PE compared to the accuracy in outpatients with suspected PE (out-of-hospital PE, OHPE), possibly due to differences in comorbidities and causes of admission [20,21,22]. Other researchers support that the probability (the “risk”) of PE can be objectified successfully in both out-of-hospital and in-hospital patients [23]. Most of these studies are retrospective in nature and/or non-consecutive patients were included [20,21,22], while a large prospective cohort study will improve our knowledge about the risk stratification and the diagnostic dilemmas in hospitalised patients with PE [24]. 

To date, only a few data studies have compared IHPE with OHPE directly, and extensively. We prospectively examined the clinical characteristics, biochemical markers, and echocardiographic indices in consecutive patients with IHPE and OHPE.

## 2. Materials and Methods

This report is part of a prospective, single-arm, single-centre study of documented PE, conducted at the University Hospital of Ioannina, a tertiary hospital in Northwestern Greece [25]. The protocol focused on echocardiographic indices and biochemical markers in PE, and it was approved by the Scientific Research Committee of the University Hospital of Ioannina [15/19-6-2019 (θ.6)]. The study complies with the Declaration of Helsinki. Informed consent was obtained from all subjects involved in the study.

### 2.1. Study Protocol and Patient Selection

Consecutive adult patients with a documented non-COVID-related thrombotic PE were included in the study. The diagnosis of PE was confirmed by computed tomography pulmonary angiography (CTPA) or a high-probability perfusion lung scan in combination with a normal chest X-ray and clinical judgement. Bedside transthoracic echocardiography (TTE) was used for diagnostic purposes in high-risk (haemodynamically unstable) patients where CTPA could not be conducted for time-saving purposes. Patients with COVID-19-related PE were excluded from the present report and defined as people who tested positive for severe acute respiratory syndrome-coronavirus-2 (SARS-CoV-2) in the previous 30 days or during hospitalisation. Patients were divided into two groups according to the conditions under which they developed PE: patients admitted to the hospital due to PE (OHPE) and patients who suffered from PE during the hospitalisation for another reason in any department of the hospital (IHPE). Patients with non-thrombotic PE were also excluded from the study.

Demographics and past medical history, including age, gender, predisposing risk factors for developing PE, comorbidities, body mass index [weight (Kg)/height (m^2^)], medication, PE-related symptoms, time from the onset of symptoms to the diagnosis, systemic arterial blood pressure, heart rate, respiratory rate, and electrocardiogram (ECG) at diagnosis, were recorded. The Geneva (original and simplified) score, the Wells criteria and score (original and simplified), and the Pulmonary Embolism Severity Index (PESI) and simplified PESI (sPESI) score were calculated in both groups [4,5,9,26,27]. The Charlson Comorbidity Index (CCI) was also calculated in all patients [28]. In the IHPE group, the cause of admission to the hospital was recorded. 

### 2.2. Biochemical Markers

High sensitivity cardiac Troponin-I (hs-cTnI, Beckman Coulter, Inc., Brea, CA, USA) and D-dimer (STA-Liatest D-Di PLUS kit using STA R Max3 and STA Compact Max3 analyzers, Diagnostica Stago S.A.S., Asnières sur Seine, France) levels were measured during the diagnostic work-up, one or more times. The reported value of hs-cTnI (pg/mL) and D-dimers (fibrinogen equivalent units per mL, FEU/mL) of the present report was the “closest” to the diagnosis, in time. BNP measurement (Quidel Triage BNP test for Beckman Coulter Access Family of Immunoassay Systems using ethylene diamine tetraacetic acid as the anticoagulant, pg/mL) was usually performed immediately after the diagnosis of PE (the testing was not performed routinely in the emergency department of the hospital). The assay’s 99th centile for cTnI is 11.6 pg/mL for women (95% CI 8.4–18.3) and 19.8 pg/mL for men (95% CI 14.0–42.9), while the expected normal level is <0.5 μg/mL for D-dimer and ≤100 pg/mL (in patients without heart failure) for BNP. 

### 2.3. Echocardiography

The details of the RV assessment using echocardiography were published elsewhere [25]. Briefly, an analytical bedside TTE with a focus on RV function was performed within the first 24 h after diagnosis by two cardiologists with a special interest in imaging techniques, in rotation. The following echocardiographic signs were considered as “conventional” markers of RV_d/po_: RV enlargement (basal dimension > 4.2 cm or mid-diameter > 3.5 cm in the 4-chamber view or RV/left ventricle basal diameter > 1 from the apical 4-champer view), dyskinesia or hypokinesia of the free right ventricular wall and McConnell sign, flattened intraventricular septum, the “60/60 sign”, tricuspid annular plane systolic excursion (TAPSE) < 16 mm and peak systolic velocity (S′) of tricuspid annulus < 9.5 cm/s [29,30,31]. A specialised cardiologist calculated the right ventricular free wall longitudinal strain (RV-FWLS) offline using the Tom Tec 2D Cardiac Performance Image Analysis (Unterschleissheim, Germany). Any value > −20% was considered as abnormal [30,31]. 

### 2.4. Follow-Up

Patients were followed up for at least 12 months after their discharge from the hospital. At 3 and 12 months, they visited a dedicated clinic or they underwent a telephone interview (most of these dates were planned during the COVID-19 pandemic). Telephone contact with their personal physicians was used in case of doubts about the reliability of the information provided by them. We recorded death from any cause, recurrent VTE events (PE or DVT), major bleeding, and the need for a new hospitalisation (for any reason). Major bleedings were defined as per the International Society of Thrombosis and Haemostasis (ISTH) definition: fatal bleeding, symptomatic bleeding in a critical area or organ, and/or bleeding with a fall in haemoglobin level of ≥2 g/dl or leading to a transfusion of ≥2 units of packed red cells or clinically relevant non-major bleeding requiring intervention or escalation of care. A hospital chart review was performed in case of a new hospitalisation.

### 2.5. Statistical Analysis

Categorical variables were presented as frequencies and percentages while the central tendency of scale variables was expressed as mean and standard deviation or as median and interquartile range depending on the normality tests that were based on the Shapiro–Wilk test and the visual inspection of the QQ plots. Pearson’s Chi-Square test was used to assess the statistical significance between the groups and all categorical outcomes after controlling for the assumptions that related to per-cell sample size sufficiency. The independent samples t-test was used for differences in the body mass index (BMI), age, and RV-FWLS, while differences regarding hscTnI, BNP, D-dimers, and time were assessed based on the Mann–Whitney test. Differences in scale measurements were graphically attributed using boxplots and differences in percentages using comparative bar charts. An alpha level of 0.05 was used to determine statistical significance unless otherwise stated for subgroup analyses; all statistical tests were 2-tailed. All analyses were carried out using the SPSS (version 26.0, IBM Corp, Armonk, NY, USA) software package. 

## 3. Results

### 3.1. Patient Characteristics

From September 2019 to March 2022, a total of 180 (84 women) consecutive patients with documented non-COVID-related PE were included in the study. The diagnosis was based on the CT scan in 168, on the perfusion lung scan in 7, and on the TTE alone in 4 patients. In one patient, the diagnosis was based on the combination of the clinical judgment and positive lower limb vein compression ultrasonography (CUS) testing. The mean age was 68.9 (±14.5) years. Eighty-nine patients (49.4%) were hospitalised for another reason when PE was diagnosed. The most common causes of admission were infections, cancer, surgical interventions and fractures, and coronary heart disease. The cause of admission for 11 patients was the investigation of a mass. Of these, 10 eventually had cancer and the under investigation mass was either the primary focus or a metastatic lesion of the cancer. Analytically, the causes of admission are shown in Table 1.

In-hospital mortality was 8.9% (16/180). Seven patients (3.9%) died due to hemodynamic instability from PE, and 9 (5%) from a complication related to their main disease or from another hospitalisation-related complication. Seventeen patients (9.4%) received intravenous thrombolysis. 

The most common strong risk factors were major trauma or surgery (27), a history of venous thromboembolism (27), and a fracture of the lower limb (8). Thirty-five patients (19.4%) had active cancer and 9 (5%) patients had a history of cured cancer. Sixty-eight patients (37.8%) had at least 1 strong risk factor, 145 (80.6%) had at least 1 moderate, and 164 (91.1%) had a weak risk factor for VTE (a patient might have more than 1 risk factor which may belong to different risk categories). Forty-three patients (23.9%) had a history of chronic lung disease or congestive heart failure. 

Regarding the symptoms at presentation, dyspnoea was the most common (106, 58.8%), in total. Forty-eight (26.7%) patients reported chest pain (pleuritic or retrosternal), 13 (7.2%) unilateral lower limb pain, 10 (5.6%) syncope, and 10 (5.6%) haemoptysis. Fever (>37.2 °C) was recorded in 31 (17.2%) patients. Forty-four (24.4%) patients were on anticoagulant treatment at the time of diagnosis (prophylactic or therapeutic dose). 

The ECG was considered as “normal” in 77 (42.8%) patients at the time of diagnosis. Sixty-eight (37.8%) patients had tachycardia (>100 beats per minute), 48 (26.7%) had negative T waves in the leads V1-V4, 25 (13.9%) had a new onset right bundle branch block (RBBB) or signs of RV strain, 16 (8.9%) had atrial fibrillation and 13 (7.2%) had the S1Q3T3 “pattern”. 

Table 2 shows the differences in demographics and clinical characteristics observed between the two groups of interest. 

Eighty-nine patients (49.4%) had PE during hospitalisation for another reason. The percentage of women was similar in the two groups (43/89 in IHPE vs. 41/91 in OHPE, *p* = 0.609). Patients in the IHPE group were on average 5 years older (71.35 ± 12.65 vs. 66.62 ± 15.75 years, *p* = 0.028), they had more frequently at least one moderate risk factor (77/89 vs. 23/91, *p* = 0.046) and cancer as a comorbidity (28/89 vs. 16/91, *p* = 0012), and they were more frequently on anticoagulants treatment at the time of the PE diagnosis (33/89 vs. 11/91, *p* < 0.001), compared with the patients with IHPE. On the other hand, more patients with a history of previous VTE belonged to the OHPE group (20/91 vs. 7/89, *p* = 0.008). No differences were observed regarding BMI (28.43 ± 5.43 in the OHPE group and 27.94 ± 6.04 in the IHPE group, *p* = 0.566). Similar percentages of patients had a history of chronic lung disease or congestive heart failure in the two groups (20/91 in the OHPE group and 23/89 in the IHPE group, *p* = 0.543). Furthermore, regarding the smoking status, the recorded percentages were similar in the 2 groups (30/91 vs. 21/89 active smokers, *p* = 0.339). The Charlson comorbidity score was significantly higher in the IHPE group [4 (4) vs. 3 (3); *p* < 0.001]. 

### 3.2. Symptoms, Signs and Clinical Scores

Time from the onset of symptoms to the diagnosis was significantly longer in the OHPE group (*p* < 0.001), as the median time was significantly higher in this group of patients [72 (216) h] compared to the IHPE group [12 (20) h]. 

More frequently in the case of OHPE, patients presented with pleuritic or retrosternal chest pain (37/91 vs. 11/89, *p* < 0.001), lower limb pain (11/91 vs. 2/89, *p* = 0.011), and haemoptysis (10/91 vs. 0/89, *p* = 0.001). The proportion of patients with other symptoms (such as dyspnoea, syncope, or cough) did not significantly differ between the two groups (Table 2). No statistically significant differences were found for fever (16/79 in the IHPE group vs. 15/91 in the OHPE group, *p* = 0.791), pathological ECG (52/79 in the IHPE group vs. 43/91 in the OHPE group, *p* = 0.133), or for the subcategories of the pathological ECG (e.g., tachycardia, negative T waves in VI through V4, or S1Q3T3 sign). 

The median Wells and Geneva scores (original or simplified) did not differ significantly in the two groups [Wells in IHPE vs. OHPE group: 4.00 (4.00) vs. 4.00 (4.50), *p* = 0.855; sWells: 2.00 (2.00) vs. 2.00 (2.00), *p* = 0.106; Geneva: 6.00 (4.00) vs. 6.00 (5.00), *p* = 0.865; sGeneva: 3.00 (2.00 vs. 3.00 (2.00), *p* = 0.104]. Moreover, the categories of scores were similar in the two groups. For example, the percentages of patients with low (14.3% of OHPE vs. 6.7% in IHPE), intermediate (70.3% vs. 79.8%), and high (15.4% vs. 13.5%) probability of PE according to the Geneva score did not differ significantly between the two groups (*p* = 0.215).

The PESI score was significantly higher in the IHPE group compared to the OHPE group [105 (46) vs. 89 (52), respectively, *p* = 0.001] In the total population, 57 (31.7%) patients had a sPESI score of 0, and 123 (68.3%) patients had a sPESI score ≥ 1, while the median sPESI score was 1 (0, 2). Scores ≥1 were less frequently observed in the OHPE group compared to the IHPE group (58.2% vs. 78.6%, *p* = 0.003).

### 3.3. Biochemical Markers

**Figure 1:**
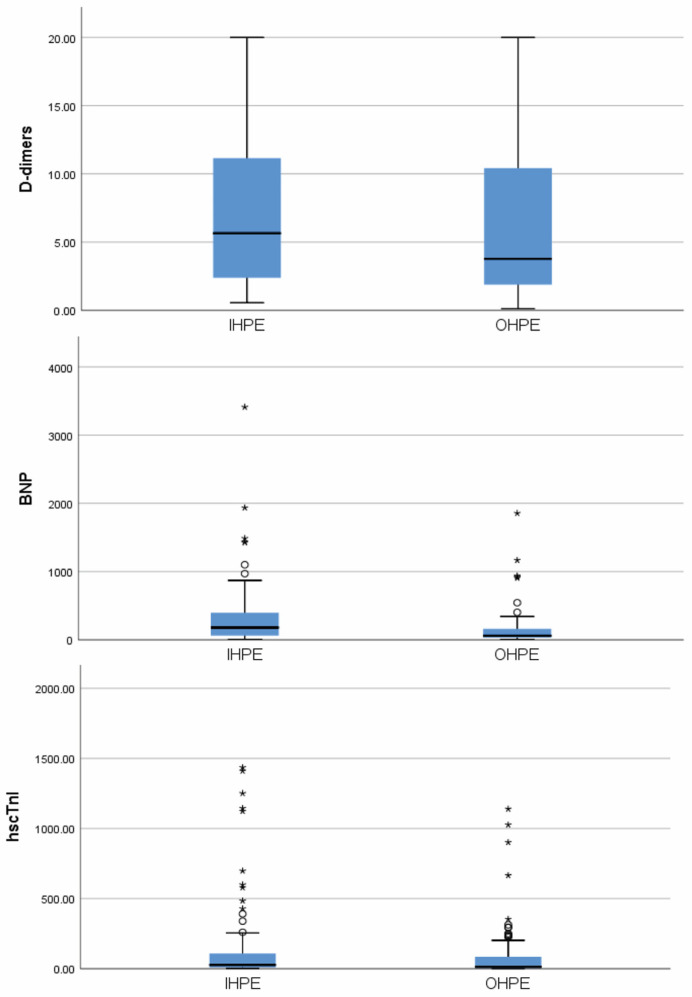
The values of hscTnI (pg/mL), BNP (pg/mL), and D-dimers (FEU/mL) in patients with in-hospital (IHPE) and out-of-hospital pulmonary embolism (OHPE). HscTnI: high sensitivity cardiac troponin I; BNP: brain natriuretic peptide; IHPE: in-hospital pulmonary embolism; OHPE: out-of-hospital pulmonary embolism.

Figure 1 shows the values of hscTnI, BNP, and D-dimer testing in the two groups of interest. 

The hscTnI and BNP values were significantly higher in the IHPE group compared to the OHPE one [26.65 (9.75, 108.80) pg/mL vs. 12.90 (4.60, 85.00) pg/mL, *p* = 0.013 and 180 (62, 396) pg/mL vs. 60 (31, 162), *p* < 0.001, respectively]. Numerically higher D-dimer values were observed in the IHPE group, but values did not differ significantly (*p* = 0.189). Significantly higher values of hscTnI, D-dimers, and BNP levels were observed in patients with sPESI score ≥ 1 than in patients with sPESI score 0 (Table S1). Moreover, patients with sPESI score ≥ 1 had significantly higher values of Wells or simplified Wells score while the Geneva scores (original or simplified) did not differ significantly (Table S1).

### 3.4. Echocardiography (Figure 2)

In 29 patients, no detailed echocardiographic study focusing on “conventional” indices of RV_d/po_ was performed because of early death (<24 h) after diagnosis due to haemodynamic instability or due to a poor echocardiographic window. Pathological conventional echocardiographic indices were recorded in 19/78 (24.4%) patients in the OHPE group and in 19/73 (26.0%) patients in the IHPE group (*p* = 0.813). Patients could have more than one pathological index per echocardiographic study. Analytically, 13 (16.7%) patients in the OHPE group had RV enlargement, 4 (5.1%) had dyskinesia or hypokinesia of the right ventricular free wall and McConnell sign, 10 (12.8%) had a flattened intraventricular septum, 3 (3.8%) had the “60/60 sign”, 4 (5.1%) had a TAPSE < 16 mm, and 2 (2.6%) had a peak systolic velocity (S′) of tricuspid annulus 9.5 cm/s). Regarding the IHPE group, 12 (16.4%) patients had RV enlargement, 4 (5.5%) had dyskinesia or hypokinesia of the free right ventricular wall and McConnell sign, 10 (13.7%) had a flattened intraventricular septum, 1 (1.4%) had the “60/60 sign”, 8 (11%) had a TAPSE < 16 mm, and 7 (9.6%) had an abnormal peak systolic velocity (S′) of the tricuspid annulus.

**Figure 2:**
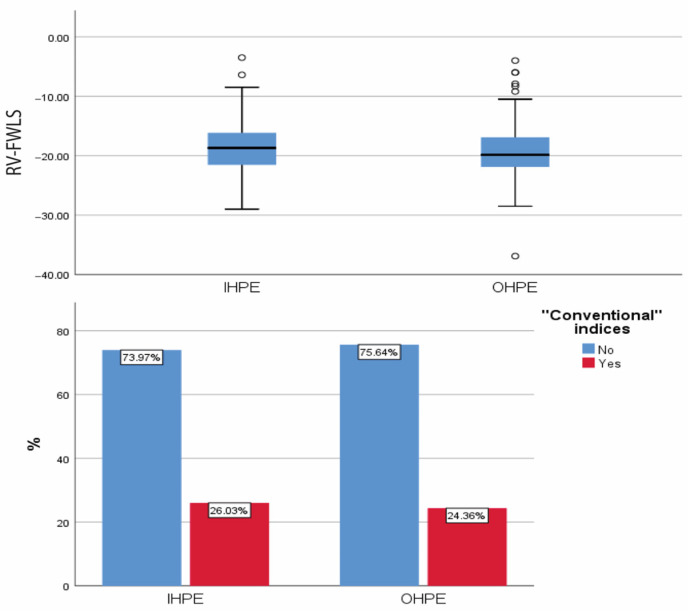
The right ventricular free wall longitudinal strain (RV-FWLS) and the existence (or not) of pathological “conventional indices” of right ventricular dysfunction or pressure overload, in percentage (%), in patients with in-hospital (IHPE) and out-of-hospital pulmonary embolism (OHPE). IHPE: in-hospital pulmonary embolism; OHPE: out-of-hospital pulmonary embolism; RV-FWLS: right ventricular free wall longitudinal strain.

One hundred and forty-three patients (76 in the OHPE and 67 in the IHPE group) had an estimation of RV-FWLS (38 patients were excluded due to early death, a poor echocardiographic window, or inadequate echocardiographic images for RV-FWLS estimation). The mean values were −19.85 (±5%) in the OHPE group and −18.7 (±5.6%) in the IHPE group (*p* = 0.211). The percentage of patients with RV-FWLS > −20% was 53.9% (41/76) in the OHPE group and 61.2% (41/67) in the IHPE group (*p* = NS).

### 3.5. Prognosis

**Figure 3:**
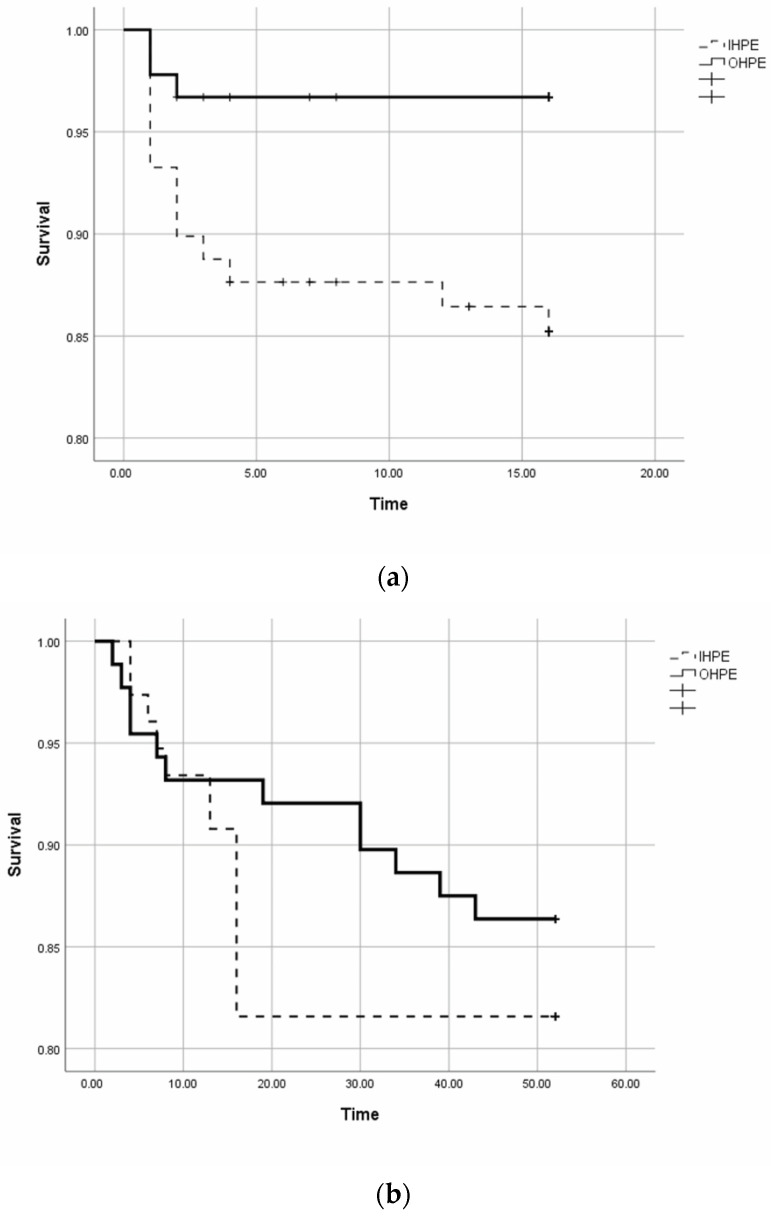
Kaplan–Meier curves show the mortality over time during hospitalisation (**a**) and post-discharge to 12 months (**b**) in patients with in-hospital (IHPE) and out-of-hospital pulmonary embolism (OHPE). IHPE: in-hospital pulmonary embolism; OHPE: out-of-hospital pulmonary embolism; Time: A in days and B in weeks.

In-hospital mortality was 3.3% (3/91) in the OHPE group and 14.6% (13/89) in the IHPE group (*p* = 0.008). Figure 3a shows the mortality over time (days) during hospitalisation.

There was a significant difference between the 2 groups (Long Rank Mantel–Cox, *p* = 0.008). The percentages of patients who received systemic thrombolytic therapy were similar in the two groups (8.8% in the OHPE group and 10.1% in the IHPE group, *p* = 0.762). 

One hundred sixty-four patients were discharged from the hospital after their hospitalisation for PE. On the day of discharge, 26 (34.2%) patients in the IHPE group (12 with cancer) and 12 (13.6%) in the OHPE group (8 with cancer) were on low molecular weight heparin and the remaining patients were on oral anticoagulation (*p* = 0.007). Ten patients (16.1% of the alive) in the IHPE group and 3 patients (4.5%) in the OHPE group stopped the anticoagulation before 12 months (*p* = 0.030). During follow-up, no one lost during the first 3 months and 2 (1.2%) patients were lost after 3 months of follow-up. The post-discharge 3-month and 12-month mortality did not differ significantly between the 2 groups. Analytically, 7 (9.2%) patients died within 3 months after discharge in the IHPE group, 6 (6.8%) in the OHPE group (*p* = 0.572), and 14 (18.4%) and 12 (13.6%), respectively, within 12 months (*p* = 0.439). The most common cause of death after discharge was cancer (12/26, 46.2%). Of these patients, four out of six in the IHPE group and 4/6 in the OHPE died due to metastatic cancer. In 4 (15.4%) patients, the cause of death was an infection, in 2 (7.7%) cardiovascular events (1 heart failure and 1 rupture aneurysm), in 1 (3.8%) ileus, and in 1 (3.8%) PE. The cause of death was not determinable in 7 (26.9%) patients. Figure 3b shows the 12-month post-discharge mortality over time (Long Rank Mantel–Cox, *p* = 0.402). Thirty-four out of the 76 patients who were discharged alive from the IHPE group and 26/88 in the OHPE group had at least one re-admission within the next 12 months (*p* = 0.044). 

Only 2 patients had a recurrent episode of VTE (both in the OHPE group) and 5 patients had major bleeding events (1 in OHPE and 4 in IHPE group) during the follow-up. Four major bleeding events (4/5) occurred between 3 and 12 months. 

## 4. Discussion

The main strength of the study is to have presented in a tertiary hospital an analytical description of the mode of presentation in consecutive patients with out-of-hospital and in-hospital PE. Our main results from the prospective comparison of these two groups were: (a) IHPE constitutes half of the PE population; (b) the 2 groups differed regarding risk factors, prior history of VTE, and symptoms. (c) IHPE patients had higher values of PESI score and higher in-hospital mortality; (d) IHPE patients have higher values of biochemical markers indicating RV dysfunction (hsTnI and BNP); and (e) the two groups had a similar proportion of abnormal echocardiographic indices of RV_d/po_. 

The aim of this study was to compare patients with PE according to the location of the disease: out-of-hospital PE versus PE occurring in already hospitalised patients. This is not only a theoretical question. In patients with suspected PE, clinical characteristics (e.g., lower limb pain, haemoptysis, beats per minute) and biomarkers (hscTnI, BNP) influence the possibility of suffering from PE. Many years ago, Schrecengost et al. examined the D-dimer testing in consecutive hospitalised patients and outpatients with suspected PE. They showed that the diagnostic accuracy of D-dimer testing in hospitalised patients with suspected PE is poor and lower than in out-of-hospital patients. They attributed their results to comorbidities and thrombosis other than to PE [20]. In another study, Miron et al. reported that D-dimer testing appears to be useless in hospitalised patients [22]. On the other hand, Posadas-Martínez et al., in a large prospective registry of patients who developed PE during hospitalisation showed that the Wells score is accurate in predicting the probability of PE in hospitalised patients. However, hospitalised patients with suspected PE had a higher possibility of suffering from the disease than outpatients with suspected PE [23]. When Söhne M, in a retrospective study, investigated the sensitivity, the negative predictive value of a non-high clinical probability of PE (based on Wells score), and a normal D-dimer result in outpatients and inpatients with suspected PE, they found that these tests were less reliable in inpatients compared to outpatients, especially in the elderly [32]. Also, the reliability of the scores for the discrimination of the risk for VTE in hospitalised patients differed among patients with or without anticoagulation treatment [33]. Furthermore, regarding the Geneva score and its revised form (revised Geneva score), it is known that both scores have been validated only in outpatients [8]. 

The aforementioned studies focused on diagnostic testing and risk scores in inpatients and outpatients with PE. Our study adds a detailed description of the mode of presentation, the biomarkers, the echocardiogram, and the prognosis in patients with IHPE and OHPE. The two groups were similar in size but many differences existed between them. Our demographic results showed an older population (about five years) in IHPE patients. Previous studies reported that advanced age is associated with a greater risk and greater mortality of VTE. We also observed a higher proportion of cancer patients in this group, and it is known that cancer is largely a disease of older age. Both age and cancer have been shown to be independently associated with poorer outcomes, and in our population, IHPE patients had higher in-hospital mortality [34,35,36]. 

The rates of VTE-related risk factors differed between the two groups. History of previous VTE and intermediate risk factors were more frequently observed in OHPE, while older age and cancer in IHPE. Interestingly, mortality after discharge was similar in the two groups. A possible explanation could be that patients with more adverse prognoses died in the hospital. Regarding symptoms, a lower percentage of chest and lower limb pain was recorded in the IHPE group, and this may be the consequence of the usage of analgesics in hospitalised patients. 

Patients in the IHPE group had worse prognostic indices than those in the OHPE group regarding PESI, sPESI scores, and CCI. The results were as expected as the IHPE group included older patients and more patients with cancer. Ten years ago, Ng AC, et al., showed that CCI following PE independently predicted in-hospital and post-discharge death [37]. Data from the Spanish National Hospital Discharge Database showed that there has been an increase in CCI values in patients with PE during the last two decades [38]. The in-hospital mortality was higher in patients suffering from more comorbidities. Zöller et al., using Swedish medical databases, showed that the higher the CCI, the higher the short-term mortality after VTE. They concluded that comorbidities are important for the risk assessment of VTE [39]. 

Sixty patients from the total population were re-admitted during the 12-month period of follow-up. Our results are in accordance with the literature. Aujesky et al. examined re-admissions during the first 30 days after PE from 186 non-Veterans Affairs acute care hospitals in Pennsylvania. They found that early re-admission is common (14.3%). Patients with higher PESI scores and those with comorbid conditions were relatively more likely to be re-admitted [40]. In our IHPE population, the patients had higher PESI scores and CCI values and more often re-admissions at 12 months than in the OHPE group. 

Biomarkers of RV dysfunction (hscTnI and BNP) were detected at higher levels in patients who belonged to the IHPE group compared with OHPE patients. These results can be attributed either to PE with a higher risk or to many other causes such as older age and cancer [41,42,43,44]. It should be noticed that patients in the IHPE group had similar echocardiographic characteristics of RV_d/po_ compared to OHPE patients despite the higher levels of hsTnI and BNP and the poorer prognosis. This finding has to do with both “conventional” indices and RV-FWLS, supporting that comorbidities (e.g., cancer) and age were the main compounding factors in the IHPE group and that the “severity” of PE (based on RV_d/po_) was the same in the 2 groups. 

Finally, the higher in-hospital mortality in hospitalised patients for other reasons suggests that alternative treatment strategies may have an indication in the case of those patients, even if PE is non-high-risk. Recent studies that examined the efficacy of interventional therapies for PE (with or without intrapulmonary local thrombolysis) have shown encouraging results. Dedicated catheters for intrapulmonary thrombolysis, ultrasound-assisted intrapulmonary thrombolysis, and aspiration thrombectomy devices have been developed for the treatment of high- or intermediate-risk PE [45]. For example, the FlowTriever all-comer registry for patient safety and hemodynamics (FLASH) evaluated the safety and effectiveness of the FlowTriever System (Inari Medical) in intermediate- and high-risk PE and reported 0% 48-h mortality and 0.3% 30-day mortality. Eighty-four percent of the patients had a sPESI score ≥ 1. Furthermore, major bleeding events appeared rarely (1.4% at 48 h). Despite the fact that this was a registry and not a randomised trial and, obviously, the patients were non-consecutive, the results were quite encouraging [46]. It would be interesting to investigate whether interventional therapies have better results compared to anticoagulation alone in IHPE patients with intermediate-risk PE. 

The study has some limitations. Firstly, it is a single-centre study, conducted at a tertiary hospital and exclusively in a Caucasian population. Secondly, the number of included patients is relatively small. Thirdly, COVID-19-related PE was an exclusion criterion. Therefore, our results and deductions cannot be referred in these patients. Fourthly, there is no control group (in-hospital or out-of-hospital patients with suspected PE and another final diagnosis). Finally, postmortem data from out-of-hospital sudden deaths are not included in our results. 

## 5. Conclusions

In a prospective cohort of consecutive patients with PE, patients who suffered from IHPE vs. OHPE differed in clinical characteristics, demographics, and co-morbidities. IHPE patients had a worse in-hospital prognosis possibly due to older age and comorbidities (mainly cancer). Results of studies on patients presented in emergency departments should be not generalised to hospitalised patients for another reason who suffer from PE. 

## Figures and Tables

**Table 1:** Causes of admission to the hospital of patients with an in-hospital diagnosis of pulmonary embolism.

Cause	N, %
Infection	27 (30.3)
Mass investigation *Final diagnosis:* *Malignant* *Nonmalignant*	11 (12.4) *10 (11.3)* *1 (1.1)*
Surgery (includes all the surgical procedures except for surgery for cancer)	11 (12.4)
Surgery for cancer	5 (5.6)
Chemotherapy session	2 (2.2)
Fracture (without surgery)	8 (9.0)
Coronary artery disease (acute or chronic coronary syndrome)	7 (7.9)
Acute heart failure	3 (3,4)
Stroke	5 (5.6)
Chronic obstructive pulmonary disease (exacerbation)	2 (2.2)
Other	8 (9.0)
**Total**	**89 (100)**

**Table 2:** Main clinical characteristics in patients with in-hospital pulmonary embolism (IHPE) and out-of-hospital pulmonary embolism (OHPE).

Clinical Characteristics		IHPE		OHPE			
		N	M (SD)	N	M (SD)	*T*	*p*
**Age (years)**		89	71.35 (12.65)	91	66.62 (15.75)	2.222	**0.028**
**BMI (kg/m^2^)**		89	27.94 (6.04)	91	28.43 (5.43)	−0.576	0.566
		**N**	**Median (IQR)**	**N**	**Median (IQR)**	***M-W***	***p***
**Time of OSTD (hours)**		89	12 (20)	91	72 (216)	6460	**<0.001**
				
		**N**	**%**	**N**	**%**	***Χ*** **_1_** **^2^**	***p***
**History of smoking**	Smoker	21	41.2%	30	58.8%	2.008	0.399
	Ex-smoker	9	50.0%	9	50.0%		
	Non-smoker	59	53.2%	52	46.8%		
**ECG**	Normal	37	43.5%	48	56.5%	2.254	0.133
	Abnormal	52	54.7%	43	45.3%		
**Fever (>37.2 °C)**	No	73	49.0%	76	51.0%	0.070	0.791
	Yes	16	51.6%	15	48.4%		
**Dyspnoea**	No	35	47.3%	39	52.7%	0.232	0.630
	Yes	54	50.9%	52	49.1%		
**Chest pain**	No	78	59.1%	54	40.9%	18.427	**0.001**
	Yes	11	22.9%	37	77.1%		
**Lower limb pain**	No	87	52.1%	80	47.9%	6.503	**0.011**
	Yes	2	15.4%	11	84.6%		
**Syncope**	No	84	49.4%	86	50.6%	0.001	0.971
	Yes	5	50.0%	5	50.0%		
**Cough**	No	83	51.2%	79	48.8%	2.077	0.150
	Yes	6	33.3%	12	66.7%		
**Haemoptysis**	No	89	52.4%	81	47.6%	10.356	**0.001**
	Yes	0	0%	10	100%		
**On anticoagulation treatment**	Νο	56	41.2%	80	58.8%	15.215	**<0.001**
	Yes	33	75.0%	11	25.0%		
**History of congestive heart failure or respiratory failure**	No	66	51.8%	71	48.2%	0.370	0.543
	Yes	23	53.5%	20	46.5%		
**Cancer**	No	61	44.9%	75	55.1%	8.849	**0.012**
	Yes	25	71.4%	10	28.6%		
	Cured	3	33.3%	6	66.7%		
**Major trauma/surgery**	Νο	68	44.7%	84	55.3%	10.012	**0.002**
	Yes	21	77.8%	6	22.2%		
**Previous VTE**	Νο	82	53.6%	71	46.4%	7.029	**0.008**
	Yes	7	25.9%	20	74.1%		
**Fracture of lower limb**	Νο	84	48.8%	88	51.2%	0.571	0.450
	Yes	5	62.5%	3	37.5%		
Strong risk factor *	No	54	48.2%	58	51.8%	1.423	0.491
	Yes	35	51.5%	33	48.5%		
Moderate risk factor *	No	12	34.3%	23	65.7%	3.994	**0.046**
	Yes	77	53.1%	68	46.9%		
Weak risk factor *	No	6	37.5%	10	62.5%	1.002	0.317
	Yes	83	50.6%	81	49.4%		
**Thrombolyis**	No	80	49.1%	83	50.9%	0.092	0.762

IHPE: in-hospital pulmonary embolism; OHPE: out-of-hospital pulmonary embolism; SD: standard deviation; IQR: interquartile range; BMI = body mass index (kg/m^2^); OSTD = onset of symptoms to diagnosis; ECG = electrocardiogram; VTE = venous thromboembolism; * At least one factor. The factors’ strength (strong, moderate, weak) is according to the splitting used on the 2019 Guidelines for Acute Pulmonary Embolism of the European Society of Cardiology [29].

## Data Availability

Data is available upon request to interested researchers.

## Supplementary Materials

The following supporting information can be downloaded at: https://www.mdpi.com/article/10.3390/jcdd11040103/s1, Table S1: Comparisons of several indices depending on simplified Pulmonary Embolism Severity Index (sPESI). FEU: Fibrinogen Equivalent Units; IQR: interquartile range.
