# Supplementary material for: In-Hospital versus Out-of-Hospital Pulmonary Embolism: Clinical Characteristics, Biochemical Markers and Echocardiographic Indices
Source: J Cardiovasc Dev Dis. 2024 Mar 28;11(4):103. doi: 10.3390/jcdd11040103 (PMC11050175; doi:10.3390/jcdd11040103)
Supplement: Supplementary file 1 [file jcdd-11-00103-s001.zip › jcdd-2905878-supplementary.pdf]

Supplementary Table S1 shows comparisons of several indices depending on simplified Pulmonary Embolism Severity Index (sPESI) in the total population

		sPESI		
		Φυσιολογικό	Παθολογικό	p
**Wells or**	Mean	3,27	4,13	**0.037**
	Median	3,00	4,50	
	IQR	4,00	4,00	
**Wells s**	Mean	1,56	2,10	**0.005**
	Median	1,00	2,00	
	IQR	1,00	2,00	
Geneva or	Mean	6,77	6,89	0.898
	Median	6,00	6,00	
	IQR	5,00	4,00	
Geneva s	Mean	2,72	3,07	0.088
	Median	3,00	3,00	
	IQR	1,00	2,00	
**Charlson Comorbidity Index**	Mean	2	5	**<0.001**
	Median	2	5	
	IQR	3	4	
**HS Troponin**	Mean	102,69	351,85	**<0.001**
	Median	8,20	29,50	
	IQR	21,70	174,60	
**d dimers**	Mean	4,53	7,97	**<0.001**
	Median	2,42	5,80	
	IQR	4,67	9,88	
**BNP**	Mean	160	276	**<0.001**
	Median	54	138	
	IQR	89	250	
